# Platelet-Rich Plasma vs. Mesenchymal Stem Cells for Lumbar Disc Degeneration: A Systematic Review and Meta-Analysis

**DOI:** 10.3390/ijms27093810

**Published:** 2026-04-24

**Authors:** Francesca Salamanna, Riccardo Ghermandi, Francesca Veronesi, Veronica Borsari, Cristiana Griffoni, Alessandro Gasbarrini, Gianluca Giavaresi

**Affiliations:** 1 Surgical Sciences and Technologies, IRCCS Istituto Ortopedico Rizzoli, Via di Barbiano 1/10, 40136 Bologna, Italy; veronica.borsari@ior.it (V.B.); gianluca.giavaresi@ior.it (G.G.); 2 Department of Spine Surgery, IRCCS Istituto Ortopedico Rizzoli, Via Pupilli 1, 40136 Bologna, Italy; riccardo.ghermandi@ior.it (R.G.); cristiana.griffoni@ior.it (C.G.); alessandro.gasbarrini@ior.it (A.G.); 3 Department of Biomedical and Neuromotor Sciences, Alma Mater Studiorum, University of Bologna, 40127 Bologna, Italy

**Keywords:** platelet-rich plasma, mesenchymal stem cells, degenerative disc disease, systematic review, meta-analysis

## Abstract

Platelet-rich plasma (PRP) and mesenchymal stem cells (MSCs) are promising regenerative treatments for lumbar degenerative disc disease (DDD), but their comparative efficacy is unclear. This systematic review and indirect meta-analysis, conducted according to PRISMA guidelines and the PICOS framework, evaluated their effects on pain, function, and safety. PubMed, Scopus, and Web of Science were systematically searched, yielding 1694 records, of which 21 studies (nine randomized controlled trials [RCTs] and 12 prospective studies) were included. Data were analyzed qualitatively and quantitatively, and risk of bias was assessed using RoB 2 and ROBINS-I. Meta-analyses of randomized controlled trials (RCTs) examined pain and disability at 6 and 12 months using a random-effects model. Indirect comparisons were performed using the Bucher method. Qualitative synthesis showed that PRP consistently reduced pain (often > 50%) and improved function, frequently outperforming corticosteroids. MSCs provided sustained benefits, with follow-up extending up to 72 months in some studies. Quantitative meta-analysis of five RCTs demonstrated that PRP significantly reduced pain at 6 months (mean difference [MD] −16.4 mm) and disability (ODI −12.7), with effects persisting at 12 months in one study. In contrast, MSCs showed a modest but significant reduction in pain (MD −4.3 mm) and minimal functional improvement. Indirect comparisons favored PRP over MSCs at 6 months. Both treatments exhibited favorable safety profiles, with mostly mild and transient adverse events. Overall, PRP appears more effective than MSCs in the short to mid-term, although both therapies are safe. Further high-quality head-to-head RCTs are needed to confirm these findings and define optimal clinical indications.

## 1. Introduction

Low back pain (LBP) remains one of the leading causes of disability worldwide, affecting millions of people and generating a significant socioeconomic burden [1,2]. Among the various etiologies of chronic LBP, lumbar degenerative disc disease (DDD) is a prevalent condition involving a multifactorial process characterized by progressive changes in the structure and function of intervertebral discs [3]. These changes occur with aging, may have a genetic predisposition, and can be exacerbated by disease and/or injury [1,2]. They may include the loss of disc height, dehydration, annular tears or fissures, and migration of nucleus pulposus content into the annulus fibrosus. These contents contain high concentrations of pro-inflammatory cytokines that initiate chemical sensitization of nociceptors located in the outer annulus fibrosus [3,4,5,6]. Other pathological processes observed during DDD include the loss of glycosaminoglycans, an increase in denatured type II collagen, and the presence of fibronectin fragments [3,4,5,6]. Over time, these degenerative changes may result in altered spinal biomechanics, instability, and compression of adjacent nerves or the spinal cord [3,4,5,6].

Diagnosis of DDD is typically based on a clinical evaluation, including a detailed history and physical examination. Imaging studies such as X-rays, magnetic resonance imaging (MRI), and computed tomography (CT) scans may be used to confirm the diagnosis and assess the extent of degeneration [7,8,9]. Once diagnosed, treatment of DDD usually begins with conservative measures, including physical therapy, non-steroidal anti-inflammatory drugs (NSAIDs), analgesics, and corticosteroid injections aimed at alleviating pain and improving function [10]. In cases where conservative treatment fails or symptoms are severe, surgical interventions, such as discectomy, laminectomy, spinal fusion, or artificial disc replacement, may be considered [10,11]. However, both conventional and surgical approaches often provide only temporary relief and fail to address the underlying disc degeneration [12].

In recent years, regenerative medicine has emerged as a promising therapeutic strategy aimed at restoring disc structure and function [12,13,14]. Two of the most extensively studied biologic interventions in this context are platelet-rich plasma (PRP) and mesenchymal stem cells (MSCs) [15,16,17,18]. PRP therapy has gained popularity across multiple medical disciplines due to its regenerative potential and ability to enhance tissue healing [16]. PRP is prepared by centrifuging whole blood to isolate the platelet-rich fraction from other components [16,17]. The resulting PRP contains a concentrated mix of platelets; growth factors, including platelet-derived growth factor (PDGF), transforming growth factor-beta (TGF-β), and vascular endothelial growth factor (VEGF); cytokines; and other bioactive molecules that work synergistically to promote tissue repair, angiogenesis, and cellular proliferation [16,17]. Upon activation at the site of injury, platelets release granules containing these bioactive substances, which stimulate cell migration, proliferation, differentiation, and extracellular matrix synthesis, thereby accelerating healing and tissue regeneration [18].

Similarly, MSCs, derived primarily from bone marrow (BMSCs) or adipose tissue (ADSCs), have shown potential for disc regeneration through anti-inflammatory and immunomodulatory effects. BMSCs regulate extracellular matrix production and reduce inflammation and apoptosis by secreting cytokines such as TGF-β, insulin-like growth factor 1 (IGF-1), and VEGF [19,20]. Compared to BMSCs, ADSCs offer similar multipotent differentiation potential, with additional advantages including easier acquisition and greater availability [21]. ADSCs can promote nucleus pulposus cell (NPC) proliferation and increase the production of essential extracellular matrix (ECM) components such as collagen and proteoglycans, thus supporting disc integrity and function [21,22,23].

Despite the growing interest in biologic therapies for lumbar DDD, several critical aspects remain insufficiently addressed. In particular, the literature lacks a clear comparison between PRP and MSC-based therapies in terms of clinical indications, disease severity, and optimal patient selection. Moreover, the potential role of combined or sequential use of these treatments has not been adequately explored. These gaps highlight the need for a more comprehensive evaluation of their relative effectiveness and clinical applicability.

Therefore, the aim of this systematic review is to critically compare the clinical effectiveness of PRP and MSC-based therapies in patients with lumbar DDD. By synthesizing evidence from prospective studies and randomized controlled trials, and performing an indirect meta-analysis where appropriate, we seek to clarify their impact on pain relief, functional outcomes, and safety, as well as to provide clinically relevant insights to guide future research and therapeutic strategies.

This systematic review aims to critically compare the clinical effectiveness of PRP and MSC injection (e.g., intradiscal, transforaminal, ganglionic) in patients with lumbar DDD. By synthesizing available evidence from prospective and RCTs, both comparative and non-comparative, we seek to determine which regenerative therapy provides superior outcomes in terms of pain relief, functional improvement, and safety. A meta-analysis will be conducted, limited to RCTs, to quantitatively pool results where appropriate, thereby informing clinical decision-making and guiding future research directions.

## 2. Materials and Methods

### 2.1. Study Design and Registration

**Figure 1:**
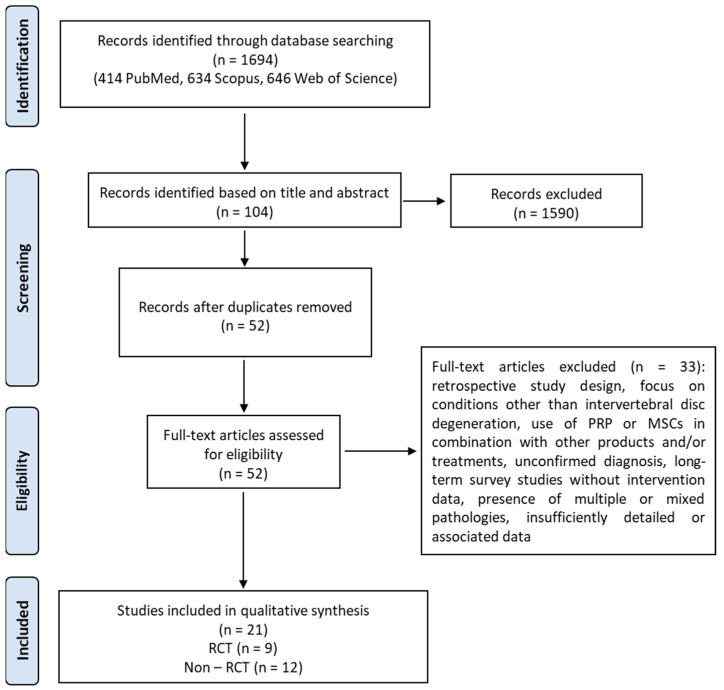
The PRISMA flow diagram for the systematic review detailing the database searches, the number of abstracts screened, and the full texts retrieved.

This systematic review was conducted in accordance with the Preferred Reporting Items for Systematic Reviews and Meta-Analyses (PRISMA) 2020 guidelines (Figure 1) [24]. The protocol was registered in the International Prospective Register of Systematic Reviews (PROSPERO) (Registration number: CRD420251075774). The detailed search strategy, including all search terms and Boolean operators used across databases, is reported in Section 2.3 and Table 1. The results of the study selection process are summarized in the PRISMA flow diagram and described in detail in Section 3.1.

**Table 1:** Development of the search strategy for PRP and MSCs in lumbar disc degeneration.

Stage	Description	Details
1	Study focus	Regenerative therapies (PRP and MSCs) for lumbar degenerative disc disease
2	PICOS framework	P: patients with lumbar DDD; I: PRP or MSCs; C: placebo or conservative treatment; O: pain and functional outcomes; S: RCTs and prospective studies
3	Research question	What is the comparative effectiveness of PRP and MSC-based therapies in lumbar DDD?
4	Controlled vocabulary	DeCS/MeSH terms: “platelet-rich plasma”, “mesenchymal stem cells”, “intervertebral disc degeneration”, “low back pain”
5	Keywords and synonyms	PRP, MSC, BMAC, ADSC, disc degeneration, discogenic pain
6	Boolean strategy	(PRP OR MSCs OR BMAC OR ADSC) AND (degenerative disc disease OR disc degeneration OR low back pain)
7	Final search strings	Detailed search queries for PubMed, Scopus, and Web of Science are reported in Table 2

**Table 2:** Search strategies used to identify studies on PRP and MSCs in lumbar disc degeneration across major databases.

PubMed	((“platelet-rich plasma”[Title/Abstract] OR PRP [Title/Abstract]) OR (“mesenchymal stem cells”[Title/Abstract] OR MSC[Title/Abstract] OR “bone marrow aspirate”[Title/Abstract] OR “adipose-derived stem cells”[Title/Abstract] OR ADSC[Title/Abstract])) AND (“degenerative disc disease”[Title/Abstract] OR “disc degeneration”[Title/Abstract] OR “intervertebral disc degeneration”[Title/Abstract] OR “intervertebral disc disease”[Title/Abstract] OR “discogenic pain”[Title/Abstract] OR “low back pain”[Title/Abstract])
Scopus	TITLE-ABS-KEY ((“platelet-rich plasma” OR PRP) OR (“mesenchymal stem cells” OR MSC OR “bone marrow aspirate” OR “adipose-derived stem cells” OR ADSC)) AND TITLE-ABS-KEY (“degenerative disc disease” OR “disc degeneration” OR “intervertebral disc degeneration” OR “intervertebral disc disease” OR “discogenic pain” OR “low back pain”)
Web of Science	TS = ((“platelet-rich plasma” OR PRP) OR (“mesenchymal stem cells” OR MSC OR “bone marrow aspirate” OR “adipose-derived stem cells” OR ADSC)) AND TS = (“degenerative disc disease” OR “disc degeneration” OR “intervertebral disc degeneration” OR “intervertebral disc disease” OR “discogenic pain” OR “low back pain”)

### 2.2. PICOS and Eligibility Criteria

The inclusion criteria were defined according to the PICOS framework, in line with PRISMA guidelines, to support a direct and/or indirect comparison of biological therapies for lumbar disc degeneration [24]. Population (P): adult patients diagnosed with lumbar DDD, discogenic low back pain, or intervertebral disc degeneration, confirmed by clinical evaluation and/or imaging. Intervention (I): intradiscal/transforaminal/ganglionic injection of PRP or MSCs, including both bone marrow-derived and adipose-derived sources. Comparison (C): both direct (head-to-head) and indirect comparisons were considered, including studies comparing PRP or MSCs to control groups such as placebo, sham procedures, or conservative treatment. Outcomes (O): primary outcomes included changes in pain intensity (e.g., VAS or NRS) and functional disability (ODI or RMDQ). Secondary outcomes included quality of life measures (e.g., SF-36), imaging-based parameters (e.g., disc height, Pfirrmann grade, Modic changes), and incidence of adverse events. Study design (S): RCTs and prospective controlled clinical studies published in English were included. Exclusion criteria comprised retrospective studies, case reports, preclinical animal or in vitro studies, and narrative reviews.

### 2.3. Information Sources and Search Strategy

The literature review involved a systematic search conducted in May 2025 on three databases: PubMed, Scopus, and Web of Science. The search strategy was designed to maximize sensitivity by including both controlled vocabulary (e.g., MeSH terms) and free-text keywords. The strategy included terms related to:

- Interventions: “platelet-rich plasma”, “PRP”, “mesenchymal stem cells”, “MSC”, “bone marrow aspirate”, “BMA”, “adipose-derived stem cells”, “ADSC”.
- Target condition: “degenerative disc disease”, “disc degeneration”, “intervertebral disc disease”, “discogenic pain”, “low back pain”.

To ensure inclusion of all relevant studies, the search combined intervention-related terms with disease-related terms using the Boolean operator OR within each category, and AND between categories. Importantly, articles were included even if they investigated only one of the biological treatments (either PRP or MSCs), allowing for a direct and/or indirect comparison of their clinical effectiveness.

Additional filters were applied to limit results to clinical studies published in English between 2015 and 2025. Reference lists of included articles and relevant reviews were also screened manually to identify further eligible studies.

To improve transparency and reproducibility, the development of the search strategy is summarized in Table 1, while the complete search strings for each database are reported in Table 2.

### 2.4. Study Selection and Data Extraction

All records identified from database searches (PubMed, Scopus, Web of Science) were imported into Zotero (Version 9.0.1_x64) reference management software. Duplicate entries were automatically identified and removed using Zotero’s duplicate detection function. Two independent reviewers then screened the titles and abstracts of all unique records for eligibility. Full-text articles were retrieved for all studies deemed potentially relevant. Disagreements during the screening process were resolved by discussion or consultation with a third reviewer. Finally, the remaining studies were established in the final stage of data extraction.

Data were independently extracted by two reviewers using a pre-defined and standardized extraction form. For each study included, the following information was collected: general study details including author and year of publication, country, study design (i.e., RCT or prospective controlled study). Regarding population characteristics, we recorded the total sample size, number of patients per treatment group, mean age and sex distribution and underlying diagnosis. Follow-up data included the total duration of follow-up, and timing of outcome assessments.

Intervention-related data included detailed information about the biological treatment administered. We extracted the treatment type, preparation method, number and volume of injections, and injection technique. When applicable, characteristics of control groups (e.g., saline, sham procedure, conservative treatment) were also recorded.

Assessed outcomes included both primary and secondary measures. Primary outcomes were pain intensity, typically measured by the VAS and/or NRS, and functional disability, assessed using the ODI and/or the RMDQ. Secondary outcomes comprised quality of life assessments (such as SF-36 or EQ-5D), radiological findings (e.g., disc height, Pfirrmann grading, Modic changes) and the rate of subsequent surgery. Finally, safety data were extracted, including the type and frequency of adverse events, as well as any reported treatment discontinuations.

Information related to study quality and risk of bias was also collected, including randomization methods (if applicable), blinding procedures (for patients, clinicians, and outcome assessors), and declarations of funding sources or conflicts of interest.

### 2.5. Risk of Bias Assessment

The risk of bias in included RCTs were assessed using Cochrane Risk of Bias Tool 2.0 (RoB 2) [25]. For non-randomized studies, the ROBINS-I tool was used [26]. Two reviewers assessed the biases independently. Any disagreements were resolved through discussion or third-party adjudication. The risk of bias was reported narratively and in tabular form.

### 2.6. Quantitative and Statistical Analysis

The quantitative synthesis was deliberately restricted to RCTs, as randomization minimizes selection bias and provides the most reliable comparative estimates of the treatment effect. Prospective non-randomized studies were reviewed qualitatively but excluded from quantitative pooling to avoid confounding and to preserve the internal validity of the meta-analysis.

All statistical analyses were performed using R version 4.5.0 (R Foundation for Statistical Computing, Vienna, Austria) and the ‘meta’ and ‘metafor’ packages [27,28,29]. Continuous outcomes were pooled as MD, as all included studies used identical measurement scales. A random-effects model based on the DerSimonian–Laird estimator was selected a priori to account for anticipated clinical heterogeneity in cell preparations and injection techniques. Heterogeneity was assessed using Cochran’s Q test and quantified with the I^2^ statistic. Values of 25%, 50%, and 75% were interpreted as low, moderate, and high heterogeneity, respectively. All pooled results are reported as MD with 95% confidence intervals. A negative value was defined to favor biologic intervention (indicating lower pain or disability). Statistical significance was set at a two-sided *p* < 0.05.

In the absence of direct comparisons between biologic interventions, an indirect comparison was planned by using the Bucher method. This approach estimates an adjusted effect size by subtracting the pooled MD of one intervention versus control from that of the other, with variances summed to derive the 95% confidence interval. Although this method preserves the internal validity of the contributing comparisons, it is inherently limited by the quality and consistency of the underlying evidence.

## 3. Results

### 3.1. Study Selection and Characteristics

The literature search was conducted in three electronic databases: PubMed, Scopus, and Web of Science. The following limits were applied: English language, exclusion of review articles, and publication date between May 2015 and May 2025. The search retrieved 414 records from PubMed, 634 from Scopus, and 646 from Web of Science. After screening titles and abstracts for relevance, 41 articles from PubMed, 38 from Scopus, and 25 from Web of Science were considered potentially eligible.

All identified articles (n = 104) were combined, and duplicates were removed, resulting in a total of 52 unique records. Full texts of these 52 articles were assessed for eligibility. Following full-text screening, 21 [30] studies met the inclusion criteria and were included in the final review.

The remaining 31 articles were excluded for the following reasons: retrospective study design, focus on conditions other than intervertebral disc degeneration, use of PRP or MSCs in combination with other products and/or treatments, unconfirmed diagnosis, long-term survey studies without intervention data, presence of multiple or mixed pathologies, and insufficiently detailed or associated data.

### 3.2. Risk of Bias Assessment

Of the 21 studies included in this systematic review, 11 were classified as non-RCTs [31,32,33,34,35,36,37,38,39,40,41] and 10 as RCTs [30,42,43,44,45,46,47,48,49,50]. However, one of the studies [44], although classified as an RCT, exhibited methodological features inconsistent with a true randomized design, including an open-label format, a small sample size, and a non-blinded crossover approach. Additionally, all placebo patients were reassigned to active treatment, eliminating a true long-term control group and introducing potential selection and performance bias. As a result, the RoB 2 tool, which is specific to RCTs, could not be applied. Instead, the study was assessed using the ROBINS-I tool, which is appropriate for non-randomized studies.

**Figure 2:**
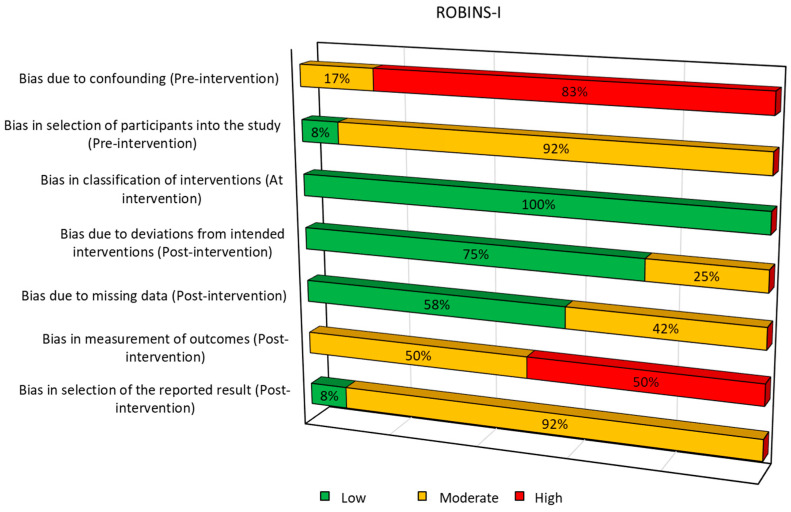
Graph summaries of risk of bias for ROBINS-I.

A total of 12 non-randomized studies were assessed using ROBINS-I (Figure 2). The risk of bias due to confounding was rated as serious in most studies (10 out of 12), indicating a limited ability to control prognostic factors. The selection of participants was consistently rated as moderate risk, while classification of the intervention was considered low risk across all studies. Bias due to deviations from intended interventions was generally moderate, as was bias due to missing data. Measurement of outcomes was often rated as serious risk [33,34,35,36,37], highlighting concerns about the reliability or objectivity of outcome assessment. Lastly, the selection of reported results showed a moderate risk in nearly all studies.

**Figure 3:**
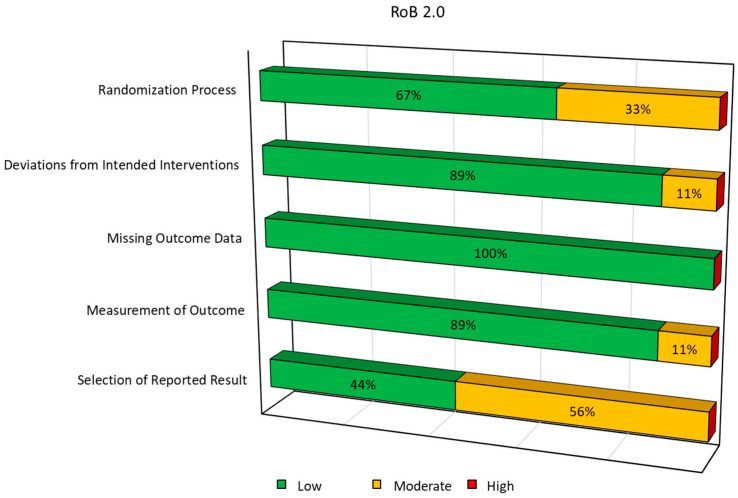
Graph summaries of risk of bias for RoB 2.0.

Nine RCTs were assessed using the RoB 2 tool (Figure 3). The randomization process was judged to be low risk in most studies (six out of nine), while some concerns were identified in three studies, mainly due to insufficient information about sequence generation or allocation concealment. Bias due to deviations from intended interventions was generally low, except for Saraf [47], which showed moderate risk. Missing outcome data was not a concern in any of the studies. Measurement of outcomes was mostly adequate, except for Saraf A et al. [47], which showed moderate risk. Finally, the selection of the reported results raised some concerns in five out of nine studies, primarily due to the absence of pre-registered protocols or pre-specified analyses.

Overall, randomized studies showed better methodological quality compared to non-randomized ones. However, some critical issues are present, particularly regarding the transparency of outcome reporting, which may affect the interpretation of findings. Non-randomized studies, while contributing relevant data, exhibited a generally moderate/high risk of bias, especially in terms of confounding and outcome measurement.

### 3.3. Data Synthesis

**Figure 4:**
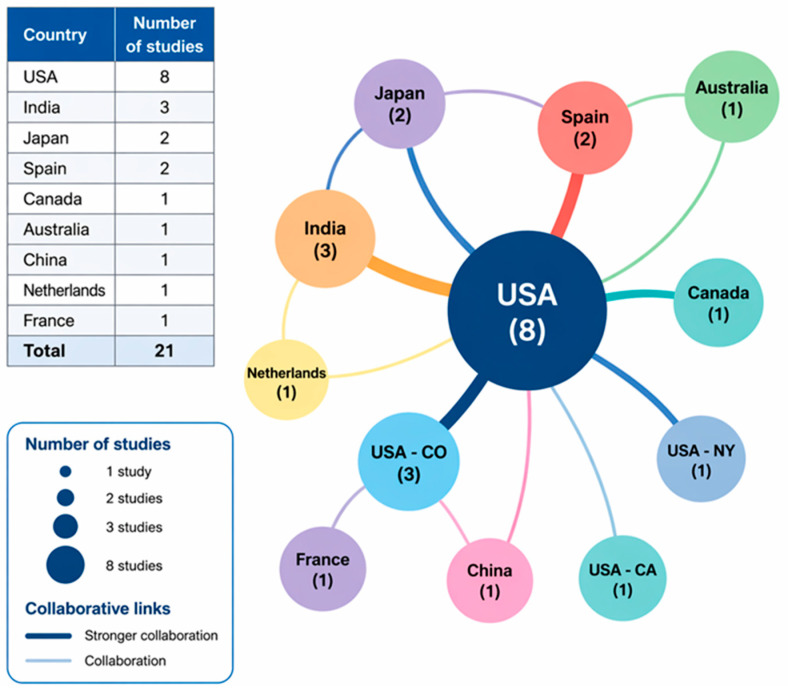
Co-authorship network among countries in studies investigating platelet-rich plasma (PRP) and mesenchymal stem cell (MSC)-based therapies for lumbar degenerative disc disease (n = 21). Node size reflects the number of studies conducted in each country, with larger nodes indicating higher research output. Lines between nodes represent collaborative relationships between countries, while thicker connections indicate stronger collaboration patterns. The network highlights the predominant contribution of the United States, along with additional contributions from Asia (Japan, China, and India), Europe (France and Spain), and other regions including Canada, Australia, and The Netherlands.

The main characteristics of the 21 studies included in this systematic review, nine RCTs and 12 prospective studies, are summarized in Table 3, Table 4 and Table 5. Geographically, the studies were conducted in diverse settings, including Japan, the United States, India, Spain, France, Canada, China, The Netherlands, and Germany (Figure 4), highlighting a global interest in regenerative therapies for DDD.

The total number of patients evaluated across all studies was 1124, of which approximately 780 received active treatment (PRP or MSC-based therapies), while 344 were assigned to control or placebo groups. Patient demographics varied across studies, with a mean age ranging between 18 and 61 years. The proportion of male participants ranged from 36% to 74%, depending on the study population. Most studies (19/21) focused on patients with chronic discogenic LBP, although a few (n = 2) included individuals with lumbar disc herniation or associated radiculopathy. The follow-up duration was highly variable, with short-term assessments conducted at 1 to 3 months in most studies, and long-term follow-up extending up to 36 or even 72 months in a few cases. The frequency of follow-up assessments also varied, with some studies including monthly evaluations during the early phase and others adopting less frequent schedules. Of the 21 studies included in the review, nine studies evaluated the efficacy of PRP injected intradiscally (n = 7) [31,36,37,41,48,49,50], transforaminal (n = 1) [47], and ganglionic (n = 1) [43]; one study investigated a specific plasma formulation, plasma rich in growth factors (PRGF) [45]. Regarding cellular products, one study assessed ADSCs [32], one study used allogeneic mesenchymal precursor cells (MPCs) [42], and one study evaluated cultured expanded BMSCs [33]. Additionally, four studies examined the use of autologous bone marrow aspirate concentrate (BMAC) [34,35,38,39]. Two studies investigated allogeneic MSCs [45,46]. Finally, only one study included a direct comparison of PRP and BMAC [44].

Overall, PRP therapy consistently showed beneficial effects in reducing pain intensity, measured predominantly by the Visual Analog Scale (VAS) or Numeric Rating Scale (NRS). Several studies reported substantial and clinically meaningful reductions in pain, typically greater than 50% compared to baseline scores. For instance, Akeda et al. [41] observed a significant reduction in VAS from 75.0 at baseline to approximately 30.0 at 12 months. In another study the same authors [50] further supported PRP efficacy, demonstrating superior outcomes compared to corticosteroids, with mean VAS scores decreasing from 68.3 to approximately 15 after 15 months of follow-up. Similar notable results were obtained by Anitua et al. [31], reporting pain reduction (NRS from 8.0 to 2.0 at 6 months) alongside improved patient functionality (ODI from 36 to 8). Additionally, Gupta A et al. [43] and Saraf A et al. [47] provided evidence of PRP’s superior clinical performance over steroidal treatments, both in pain reduction and functional improvement, reflected by significant ODI decreases (from 57.3% to 33.3%) [37]. Other PRP studies [36,40,44,49] confirmed these positive outcomes, though with variability regarding the magnitude and duration of effectiveness.

MSC-based therapies, particularly those derived from bone marrow, also demonstrated robust clinical benefits. In studies by Pettine KA et al. [38,39], intradiscal BMAC injections led to profound pain relief, with VAS reductions from approximately 82 to 22, sustained up to 36 months. Beall et al. [42], employing allogenic MPCs, confirmed sustained pain improvements versus placebo, persisting at 36 months. Centeno C et al. [33] provided evidence of long-term effectiveness, maintaining pain relief for up to six years, albeit with some variation over time. Though less frequently studied, ADSC treatments also indicated promising results. Bates D et al. [32] found that intradiscal injections of autologous ADSCs led to clinically relevant pain reduction and functional improvements in approximately two-thirds of the patients at 12-month follow-up, with average ODI score improvements around 39%. The only comparative trial identified in the systematic review is that of Navani A et al., which, although defined as an RCT, exhibited methodological features inconsistent with a true randomized design [44]. Nevertheless, it demonstrated that both PRP and BMAC intradiscal injections significantly improved pain and function compared to placebo. All placebo patients crossed over to active treatment due to insufficient pain relief. No adverse events, hospitalizations, or surgeries were reported up to 12 months. In these studies on MSC-based therapies, improvements in functional disability, assessed primarily through the ODI or Roland–Morris Disability Questionnaire (RMDQ), were consistently reported across treatment modalities. The functional gains observed were generally significant, reinforcing the overall therapeutic value of these regenerative therapies.

Radiological outcomes, including MRI-based assessments of disc height and Pfirrmann grading, were inconsistently reported across all studies on both PRP- and MSC-based therapies, with minimal or no structural improvement observed despite substantial symptomatic relief. Similar quality of life improvements, evaluated through validated scales, e.g., Short form-12 (SF-12), SF-36, and EuroQol-5D (EQ-5D), were observed consistently, further highlighting the multidimensional benefits of PRP- and MSC-based therapies. However, the magnitude and consistency of these quality of life improvements varied between studies and across follow-up intervals.

In terms of safety, both regenerative interventions demonstrated favorable profiles overall. Most studies reported minor, transient adverse events, such as localized pain or mild sensory alterations post-procedure. Serious adverse events were exceedingly rare; however, Schepers MO et al. [48] documented one isolated case of spondylodiscitis following intradiscal PRP injection, underscoring the need for meticulous procedural asepsis and standardized injection techniques.

Despite the promising results, variability in treatment protocols, including differences in PRP preparation methods, MSC sources, cell concentrations, volumes injected, and injection techniques, presented challenges in directly comparing therapeutic efficacy across studies. Furthermore, variations in patient demographics, baseline-disc degeneration severity, and follow-up durations contributed additional heterogeneity, highlighting the need for standardized, high-quality randomized controlled trials to better delineate optimal protocols and to definitively compare effectiveness among regenerative approaches.

### 3.4. Quantitative Analysis

Although nine RCTs met the overall inclusion criteria, only five [42,45,46,47,50] were meta-analyzed: three PRP RCTs [43,48,49] did not provide mean ± SD or any convertible measure of variance at the prespecified 6- and 12-month time points, and one study [30] lacked a parallel control arm, precluding calculation of a comparative effect size.

#### 3.4.1. Pain Outcomes

**Figure 5:**
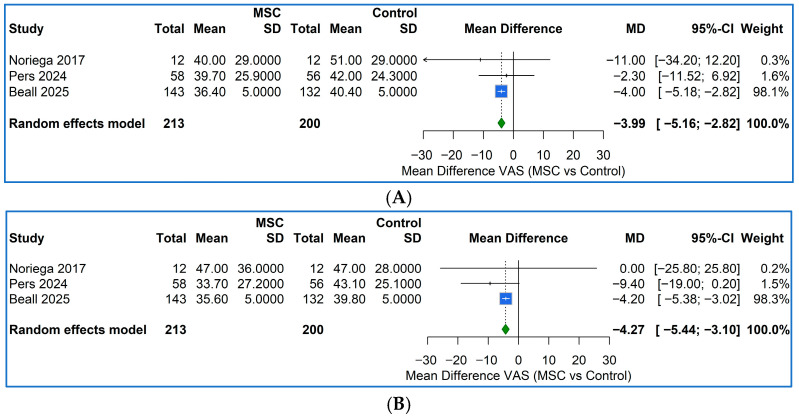
Forest plots of meta-analysis comparing MSC therapy to Control for VAS pain score at 6 months (**A**) and 12 months (**B**) post-intervention. The pooled MD with 95% confidence intervals (CIs) is shown; negative MD values favor MSC treatment. The vertical dashed line indicates no difference (MD = 0). Heterogeneity was low and not statistically significant at both 6 (Q = 0.48, *p* = 0.79) and 12 months (Q = 1.21, *p* = 0.54), supporting the robustness and consistency of the pooled VAS estimates. Noriega 2017 [45]; Pers 2024 [46]; Beall 2025 [42].

The two pooled PRP RCTs [47,50] at 6 months produced a consistent and clinically important reduction in pain: the random-effects mean difference (MD) was −16.4 mm 95%CI [−22.3, −10.5] on a 0–100 mm VAS/NRS. Across the three MSC RCTs [42,45,46], two on allogenic MSCs and one on MPC, the pooled MD favored the cellular product indicating a modest but statistically significant benefit (z = −6.67, *p* < 0.001) (Figure 5A).

At 12 months, the benefit of PRP observed in Akeda K et al. [50]—the only trial with long-term follow-up—remained essentially unchanged, with the PRP group showing a mean VAS score 8.7 points lower than the corticosteroid group (MD: −8.7 mm, 95% CI [−27.6, 10.2]). However, the confidence interval is wide and includes zero, indicating that this difference is not statistically significant. In contrast, the MSCs showed a slightly larger effect than at 6 months with a pooled MD: −4.3 mm, 95%CI [−5.4, −3.1]) (Figure 5B).

No RCTs directly comparing PRP with MSCs were identified. Therefore, an indirect comparison using the Bucher method was performed based on the pooled effect estimates from available studies. This analysis showed that, at 6 months, PRP was associated with a significantly greater reduction in pain compared to MSCs (MD −12.4 mm, 95% CI [−18.4, −6.4]). At 12 months, the difference favored PRP as well (MD: −4.4 mm, 95% CI [−23.3, 14.5]), but the confidence interval was wide and crossed zero, indicating that the difference was not statistically significant at this time point.

#### 3.4.2. Functional Disability

For ODI, the two PRP RCTs [47,50] demonstrated a pooled reduction in disability of −12.7 points (95% CI: −17.4 to −8.1) at 6 months. At 12 months, only Akeda K et al. 2022 [50] reported data, showing a reduction of −10.6 points (95% CI: −22.4 to 1.2). In contrast, the only MSC trial with available ODI data [46] showed a non-significant change of −3.9 points (95% CI: −9.5 to 1.7) at 6 months and −4.3 points (95% CI: −10.3 to 1.7) at 12 months.

In summary, this meta-analysis of five RCTs demonstrates that intradiscal PRP provides a robust and clinically meaningful reduction in pain (approximately 16 mm on a 0–100 mm scale) and disability (about 13 ODI points) at 6 months, with benefits persisting through 12 months in the single trial with long-term follow-up. In comparison, MSC therapy confers a smaller, yet statistically significant, analgesic effect (approximately 4–5 mm) and a negligible, non-significant impact on disability. Indirect adjusted analyses further favor PRP over MSCs, particularly at the 6-month time point; however, confirmation from direct head-to-head trials remains necessary.

## 4. Discussion

**Figure 6:**
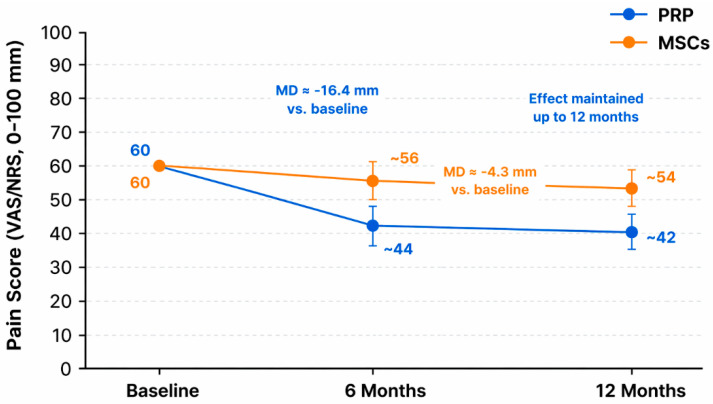
Schematic representation of pain score trends over time following PRP and MSC-based therapies for lumbar DDD. Pain scores (VAS/NRS, 0–100 mm) are shown at baseline, 6 months, and 12 months. Values are approximated from the pooled findings of the studies included. PRP demonstrated a greater reduction in pain at 6 months (mean difference ≈ −16.4 mm vs. baseline) with effects maintained at 12 months, whereas MSCs showed a more modest improvement (mean difference ≈ −4.3 mm vs. baseline). Error bars represent approximate 95% confidence intervals.

This systematic review and meta-analysis aimed to compare the clinical efficacy of PRP and MSC-based therapies in patients with lumbar DDD. The findings highlight promising, yet different, therapeutic profiles for both biologic strategies. Our meta-analysis demonstrates that intradiscal PRP provides a clinically meaningful reduction in pain (approximately 16 mm on a 0–100 mm VAS/NRS) and disability (around 13 ODI points) at 6 months, with benefits persisting at 12 months in the only trial with long-term follow-up. In contrast, MSC-based therapies yielded a smaller, though statistically significant, analgesic effect (about 4–5 mm) and negligible improvement in disability. These findings suggest that PRP may offer more rapid and pronounced symptom relief compared to MSCs in the short term. To provide a clearer and more intuitive comparison of clinical outcomes, a schematic representation of pain score trends over time following PRP and MSC-based therapies is shown in Figure 6.

However, the absence of direct head-to-head RCTs between PRP and MSCs limits the certainty of these conclusions, as indirect comparisons are susceptible to bias from heterogeneity across studies. Moreover, the limited number of trials and variations in PRP preparation, MSC sources, and outcome measures highlight the need for standardized protocols and robust, long-term comparative studies.

The observed efficacy of PRP in our review aligns with previous meta-analyses and narrative reviews reporting significant improvements in discogenic pain and disability, particularly in younger patients and in early-stage DDD [51,52,53]. A previous systematic review of intradiscal PRP injections demonstrates statistically significant VAS pain reduction (from 69.7 mm to 43.3 mm), supporting efficacy and a favorable safety profile [51]. Similarly, another meta-analysis of intradiscal PRP for discogenic low back pain showed significant pain relief at 2–6 months (Standardized Mean Difference SMD ≈ −1.43) and functional improvement (ODI SMD ≈ −0.96 at 6 months), indicating PRP’s rapid therapeutic effect [52]. PRP’s mechanism, driven by growth factors, cytokines, and anti-inflammatory mediators, may explain its rapid onset of symptom relief and neuroregulatory effects [53].

Differently, MSC therapies, while biologically compelling due to their regenerative, anti-inflammatory, and immunomodulatory capacities, have shown more variable clinical responses [52,53,54]. The slower integration and differentiation potential of MSCs may delay their symptomatic impact. Additionally, the modest effect on disability suggests that MSCs alone may not fully restore functional integrity in patients with advanced structural disc degeneration. In this context, it is important to underline that our analysis was limited to a subset of RCTs due to incomplete or non-comparable data reporting, highlighting a pervasive issue in biologic trials: a lack of standardized outcome measures and reporting formats. Furthermore, the heterogeneity in MSC source (bone marrow vs. adipose), preparation (concentrated vs. cultured, mononuclear fraction), delivery protocols, and patient selection likely contributed to the wide range of outcomes and complicates direct comparisons. An additional source of heterogeneity that warrants consideration is the origin of the biological products. PRP is consistently administered as an autologous product, whereas MSC-based therapies may involve either autologous or allogeneic sources. This discrepancy introduces potential immunological confounding factors, including differences in host response and immunogenicity, which may influence treatment outcomes and bias indirect comparisons between the two modalities. A subgroup analysis comparing autologous PRP and autologous MSCs would be valuable to minimize these confounders. However, such an analysis was not feasible in the present review due to the limited number of included studies and the lack of consistent reporting regarding the cellular source across trials. Moreover, performing such a subgroup analysis would have resulted in small and statistically underpowered groups, potentially leading to unreliable conclusions. This heterogeneity is consistent with the previous literature. Richardson and colleagues [55] highlighted methodological inconsistencies across MSC trials, including differences in donor tissue, isolation, expansion, and delivery, which may contribute to delayed or variable clinical effects and modest functional gains. Similarly, a meta-analysis by Zhang et al. [56] reported significant pain reduction with MSC injections but extreme heterogeneity (I^2^ = 98%), emphasizing inconsistencies across sources, doses, and protocols. In contrast, PRP studies included in this systematic review were more homogeneous regarding administration route (mostly intradiscal), and showed negligible statistical heterogeneity, increasing confidence in the pooled estimate.

The consistent pain and functional benefits observed with PRP suggest it may be a more accessible, cost-effective, and safer biologic alternative in early or moderate DDD, especially when surgery is not indicated. PRP’s autologous nature, minimal manipulation, and favorable safety profile may facilitate broader clinical application with lower regulatory burden compared to cell-based therapies.

In contrast, while MSCs appear safe and may offer benefits in selected cases, particularly those with more advanced degeneration or structural loss, the current data does not support widespread use outside of controlled research settings.

From a clinical standpoint, a more precise definition of indications and patient selection criteria for PRP and MSC-based therapies is still lacking. Based on the available evidence, PRP may be more appropriate for patients with early or moderate stages of disc degeneration, where inflammatory mechanisms are predominant and a rapid symptomatic effect is desirable. Conversely, MSC-based therapies, due to their regenerative and immunomodulatory potential, may be better suited for more advanced or chronic stages of the disease, although clinical responses appear more heterogeneous and often delayed.

Regarding contraindications, both approaches share general limitations, including active infections, malignancies, or severe systemic conditions. However, MSC-based therapies may present additional challenges related to cell manipulation, regulatory requirements, and higher costs, potentially limiting their broader clinical applicability compared to PRP.

Disease duration may also influence treatment outcomes. PRP appears to provide more consistent short-term benefits, while MSCs may offer longer-lasting effects in selected cases, although robust comparative evidence is still lacking.

Finally, the potential for combined or sequential use of PRP and MSCs represents an emerging area of interest. Preclinical evidence suggests that PRP-derived growth factors may enhance MSC survival, proliferation, and differentiation, potentially leading to synergistic regenerative effects. However, clinical data supporting combined approaches in lumbar DDD are currently limited and warrant further investigation.

Several limitations temper the interpretation of these findings: (1) no head-to-head RCTs comparing PRP and MSCs were available, necessitating indirect statistical inference; (2) some eligible RCTs were excluded from meta-analysis due to missing variance data, potentially biasing the quantitative synthesis; (3) variability in MSC type, dose, processing, and administration route complicates the generalizability of results; (4) not all studies reported long-term outcomes, limiting conclusions on the durability of effect, especially for MSCs.

There is an urgent need for well-designed, head-to-head RCTs directly comparing PRP and MSCs using standardized protocols, validated outcome measures, and sufficient follow-up. Additionally, efforts should be made to stratify patients by DDD severity, age, and radiological features to better identify which subgroups are most likely to benefit from each therapy. Future research should also aim to assess structural regeneration objectively through imaging, alongside symptomatic and quality of life metrics.

## 5. Conclusions

In conclusion, both PRP and MSC therapies demonstrate potential in managing discogenic low back pain; however, PRP currently offers more robust and consistent short-term clinical benefits in terms of both pain reduction and functional improvement. While MSCs remain a promising avenue, further high-quality comparative trials are needed to define their role, optimize protocols, and determine their long-term efficacy relative to PRP and other biologics.

## Figures and Tables

**Table 3:** Patient demographics and study characteristics.

Author (Year)	Country	Study Design	Total Patients (n)	Mean Age (±SD)	% Male	Diagnosis	Follow-Up (mos)
[41]	Japan	Prospective single-arm trial	14	33.8 ± 8.6	57%	Discogenic LBP	1, 2, 4, 6, 8, 10, 12
[50]	Japan	RCT	16	32.2 ± 8.3	68%	Discogenic LBP	1, 2, 3, 4, 5, 8, 15
[31]	Spain	Prospective single-arm trial	32	54.9 ± 10.1	59%	Discogenic LBP	1, 6, 12
[32]	Australia	Prospective single-arm trial	9	40.1 ± 10.3	62%	Discogenic LBP	1, 3, 6, 9, 12
[42]	OK, USA	RCT	404	42.8 ± 10.9	57%	Discogenic LBP	1, 3, 6, 12, 18, 24, 36
[33]	CO, USA	Prospective single-arm trial	33	40.3 ± 14.2	64%	Discogenic LBP	1, 3, 6, 12, 18, 24, 36, 48, 60, 72
[34]	Canada	Prospective trial	13	Median: 33–78 (63)	54%	Discogenic LBP	1, 3, 6, 9, 12
[48]	India	RCT	46	PRP: 40.64 Control: 38.92	PRP: 61% Control:74%	Prolapse/herniation lumbar IVD	1 wk, 3 wks, 6 wks
[35]	VA, USA	Prospective single-arm trial	32	45.9 ± 12.3	56%	Discogenic LBP	12
[36]	India	Prospective single-arm trial	20	34.75 ± 10.15	60%	Discogenic LBP	3, 6
[37]	VA, USA	Prospective trial	22	Median: 47.5	45%	Discogenic LBP	1, 2, 6
[44]	Campbell, CA	RCT	43	45.3 ± 9.4	45%	Discogenic LBP	1, 3, 6, 12
[45]	Spain	RCT	24	38 ± 2 (SE)	71%	Discogenic LBP	3, 6, 12
[46]	France, Spain, Italy, Germany	RCT	114	40.9 ± 8.89	65%	Discogenic LBP	1, 3, 6, 12, 24
[38]	CO, USA	Prospective two-arm trial	26	18–61 years (median 40)	42%	Discogenic LBP	3, 6, 12, 24
[39]	CO, USA	Prospective two-arm trial	26	18–61 years (median 40)	42%	Discogenic LBP	3, 6, 12, 24
[47]	India	RCT	60	PRP: 42.03 ± 11.31 Steroid: 45.83 ± 12.35	PRP: 51% Steroid: 51%	Radiculopathy, secondary to posterolateral herniated disc	1, 3, 6
[48]	Netherlands	RCT	89	PRP: 40.3 ± 10.4 Saline: 39.1 ± 11.5	PRP: 36% Saline: 42%	Discogenic LBP	1 wk, 1, 2, 6, 9, 12
[49]	NY, USA	RCT	47	PRP: 41.4 ± 8.1 Contrast agent: 43.8 ± 8.9	PRP: 48% Contrast agent: 16%	Discogenic LBP	1 wk, 1, 2, 6, 12
[40]	China	Prospective single-arm trial	31	53.4 ± 8.5	39%	Discogenic LBP	1 wk, 1, 2, 3, 6, 12
[30]	CA, USA	RCT	26	NR	46%	Discogenic LBP	1, 2

Abbreviations: RCT: randomized clinical trial; LBP: low back pain; IVD: intervertebral disc.

**Table 4:** Treatment characteristics, delivery methods, outcomes, and safety profiles.

Ref.	Group and Treatment Type	Preparation Type (PRP/MSC)	Volume (mL) and N. of Injections	Injection Technique	VAS/NRS/NPS (Baseline/FU)	RDQ/ RMDQ (Baseline/FU)	ODI (Baseline/FU)	Adverse Events
[41]	PRP	PRP releasate, isolated from clotted PRP	2 mL 1 inj. 2 inj. (only in 1 patient)	Intradiscal injection	VAS Baseline: 7.5 ± 1.3 4 wks: 3.1 ± 2.5 8 wks: 3.2 ± 2.0 16 wks: 3.4 ± 1.9 24 wks: 3.2 ± 2.4 32 wks: 3.0 ± 1.9 40 wks:2.8 ± 2.6 48 wks: 2.9 ± 2.8	Baseline: 12.6 ± 4.1 4 wks: 5.1 ± 5.2 8 wks: 5.2 ± 5.5 16 wks: 4.7 ± 4.5 24 wks: 3.6 ± 4.5 32 wks: 4.0 ± 3.0 40 wks: 3.8 ± 5.3 48 wks: 2.8 ± 3.9	-	Leg numbness (n = 2)
[50]	PRP corticosteroid (CS)	PRP releasate, isolated from clotted PRP	2 mL 1 inj.	Intradiscal injection	VAS PRP: Baseline: 68.3 ± 13.3 Mean change at: 4 wks: −19.0 ± 21.3 8 wks: −30.9 ± 22.7 12 wks: −38.3 ± 19.6 16 wks: −47.9 ± 21.2 20 wks: −45.4 ± 26.3 32 wks: −56.8 ± 20.2 60 wks: −53.4 ± 24.7 CS: Baseline: 59.4 ± 12.4 Mean change at: 4 wks: −34.9 ± 20.1 8 wks: −26.3 ± 29.8 12 wks: −32.8 ± 13.4 16 wks: −33.3 ± 13.4 20 wks: −29.8 ± 12.8 32 wks: −36.8 ± 17.1 60 wks: −36.4 ± 23.7	PRP: Baseline: 8.6 ± 4.8 Mean change at: 4 wks: −2.2 ± 5.9 8 wks: −3.4 ± 6.7 12 wks: −6.9 ± 6.4 16 wks: −6.6 ± 6.1 20 wks: −6.7 ± 6.2 32 wks: −8.5 ± 5.3 60 wks: −8.8 ± 5.0 CS: Baseline: 9.3 ± 4.7 Mean change at: 4 wks: −2.3 ± 4.2 8 wks: −1.7 ± 4.2 12 wks: −1.6 ± 3.6 16 wks: −1.7 ± 2.9 20 wks: −2.3 ± 4.6 32 wks: −3.4 ± 4.0 60 wks: −4.2 ± 4.5	PRP: Baseline: 36.0 ± 11.8% Mean change at: 4 wks: −8.2 ± 9.5 8 wks: −14.5 ± 11.6 12 wks: −17.9 ± 13.2 16 wks: −23.6 ± 14.9 20 wks: −21.9 ± 13.4 32 wks: −26.9 ± 13.1 60 wks: −26.6 ± 14.8 CS: Baseline: 33.3 ± 11.6% Mean change at: 4 wks: −7.2 ± 8.4 8 wks: −7.7 ± 8.9 12 wks: −11.2 ± 7.8 16 wks: −11.9 ± 7.3 20 wks: −12.7 ± 6.1 32 wks: −14.5 ± 10.8 60 wks: −13.9 ± 9.7	Post-injection pain (n = 1, PRP) Muscle weakness (n = 1 PRP, n = 1 CS)
[31]	PRGF	Activation of PRGF with the addition of PRGF activator (10% calcium chloride) at a ratio of 20 μL of PRGF activator per mL of PRGF	3 mL 2–3 inj.	Intradiscal injection	NRS Baseline 8.0 [6.3–8.8] 1 mos 3.0 [1.3–5.0] 3 mos 2.5 [1.0–4.0] 6 mos 2.0 [0.0–3.0]	-	Baseline 36 [28–50] 1 mos 12 [3–23] 3 mos 6 [0–16] 6 mos 8 [2–16]	Slight sensory alteration (n = 2)
[32]	ADSCs	Autologous abdominal liposuction to isolate and expand ADSCs	1 mL (10 × 10^6^ ADSCs) 2 inj. (at baseline and at 6 mos FU)	Intradiscal injection	NRS At both 6 and 12 mos, 5/9 participants reported improvements ≥50% in their average pain score. Two participants reverted to baseline average pain levels	-	6 months: 100% (n = 9) improved from baseline Improvement range: 4–80% Mean improvement: 34% 67% (n = 6) ≥30% improvement 12 months: 89% (n = 8) improved from baseline Improvement range: 8–93% Mean improvement: 39% 11% (n = 1) returned to baseline 67% (n = 6) ≥30% improvement 56% (n = 5) shifted to milder ODI categories: 2: severe → moderate 3: moderate → minimal	Mild pain (n = 7), minimal discharge and/or mild bruising (n = 8) following lipoharvest procedure and n = 1 experienced moderate pain
[42]	Allogenic mesenchymal precursor cells (MPC) MPC + HA Saline control	MPCs in saline; ~6 million MPCs in 1% HA [MPC + HA]; Saline	2 mL 1 inj.	Intradiscal injection	VAS Baseline MPCs: 60.3 ± 12.91 MPCs + HA: 60.4 ± 13.00 Control group: 57.1 ± 13.56 6 mos MPCs: 36.4 ± 5.25 MPCs + HA: 33.9 ± 5.35 Control group: 40.4 ± 5.61 12 mos MPCs: 35.6 ± 5.24 MPCs + HA: 31.4 ± 5.33 Control group: 39.8 ± 5.37 24 mos MPCs: 38.0 ± 5.60 MPCs + HA: 33.0 ± 5.39 Control group: 40.6 ± 5.55 36 mos MPCs: 35.9 ± 5.20 MPCs + HA: 33.8 ± 5.86 Control group: 39.8 ± 5.80	-	Baseline MPCs: 41.73 ± 9.84 MPCs + HA: 41.25 ± 10.51 Control group: 42.23 ± 10.59 ODI improved over time for all treatments compared to baseline without significant differences	Most common TEAEs: Back pain (MPC: 36.4%, MPC + HA: 43.8%, Control: 40.0%) Pain in extremity (13.6%, 13.3%, 14.6%) Arthralgia (9.3%, 12.5%, 10.0%) - More treatment-related TEAEs in MPC/MPC + HA (mainly transient back pain, resolved in 30–45 days) - No treatment-related SAEs - PTIs at treated level: MPC 11.0%, MPC + HA 10.5%, Control 11.1% - <10% neurologic deterioration in all groups
[33]	Cultured-expanded bone marrow mesenchymal stem cells (BMSCs)	BMSCs (range 1.73 × 10^6^–4.5 × 10^7^)	1–3 mL 2 inj.	Intradiscal injection	NPS (Mean) Baseline: 5.2 1 mos: 1.5 3 mos: 1.6 6 mos: 1.4 12 mos: 0.6 18 mos: 2 24 mos: 1.2 3 yrs: 2 4 yrs: 2.5 5 yrs: 3.7 6 yrs: 3.3	-	-	Pain (n = 3) Large, herniated nucleus pulposus (n = 1)
[34]	Autologous bone marrow aspirate concentrate (BMAC)	Volume of BMAC injected per disc based on the intradiscal pressure created during injection	1–6 mL 1 inj.	Intradiscal injection	VAS Baseline: 6 ± 1.87 1 mos: 3 ± 1.78 3 mos: 3.15 ± 1.91 6 mos: 3.38 ± 1.76 9 mos: 2.23 ± 1.48 12 mos: 2.23 ± 1.30	-	-	Pain
[43]	PRP Steroid	Standard PRP preparation kit	2 mL of PRP 0.5 mL of 0.5% bupivacaine 1 inj.	Ganglionic injection	VAS (Mean) PRP: Baseline: 75 1 week: 55 3 wks: 35 6 wks: 20 Steroid (Mean): Baseline: 75 1 week: 35 3 wks: 30 6 wks: 40	-	PRP (Mean): Baseline: 60 1 week: 50 3 wks: 35 6 wks: 25 Steroid (Mean): Baseline: 60 1 week: 40 3 wks: 35 6 wks: 35	None
[35]	Autologous BMAC	BMAC from posterior iliac crest	3 ± 0.4 mL per level 1 inj.	Intradiscal injection	VAS back Baseline: 5.4 ± 2.3 12 mos: 3.0 VAS leg Baseline: 2.8 ± 2.5 12 mos: 1.3	-	Baseline: 33.5 ± 13.6 12 mos: 21.1	None
[36]	PRP	PRP obtained by two-spin technique	1.69 ± 0.32 mL per level 1 inj.	Intradiscal injection	NRS Baseline: 5.85 ± 1.14 3 mos: 4.55 ≈ ±1.5 6 mos: 3.1 ≈ ±1.3	-	Baseline: 35.7 ± 7.74 3 mos: 24.8 ≈ ±7.5 6 mos: 18.6 ≈ ±6.5	None
[37]	PRP	NR	1.5 mL 1 or 2 inj.	Intradiscal injection	VAS (Median IQR) Baseline: 64.5 (55–75) 1 mos: 48 (26–66) 2 mos: 41.5 (22–62) 6 mos: 48 (17–65)	-	(Median IQR) Baseline: 31.5 (26–40) 1 mos: 27.5 (18–36) 2 mos: 22.5 (34–14) 6 mos: 22 (10–30)	Severe pain exacerbation (n = 1)
[44]	Autologous PRP or BMAC Placebo (saline)	Commercial PRP system Commercial BMAC system	1–2 mL 1 inj.	Intradiscal injection	NRS Placebo 1 mos: 6.2 ± 1.1 3 mos: 5.8 ± 1.0 6 mos: 5.6 ± 0.9 12 mos: 5.3 ± 0.8 PRP 1 mos: 4.4 ± 1.0 3 mos: 3.4 ± 1.0 6 mos: 2.9 ± 0.9 12 mos: 2.4 ± 0.9 BMC 1 mos: 4.5 ± 1.0 3 mos: 3.1 ± 1.0 6 mos: 2.6 ± 0.8 12 mos: 2.2 ± 0.8	-	Placebo Baseline: 48% ± 5% 1 mos: 46% ± 5% 3 mos: 44% ± 5% 6 mos: 43% ± 5% 12 mos: 41% ± 5% PRP Baseline: 47% ± 5% 1 mos: 38% ± 4% 3 mos: 34% ± 4% 6 mos: 27% ± 4% 12 mos: 24% ± 4% BMC Baseline: 48% ± 5% 1 mos: 36% ± 4% 3 mos: 32% ± 4% 6 mos: 27% ± 4% 12 mos: 31% ± 5%	Temporary LBP related to disc injection
[45]	Allogenic MSCs Placebo (mepivacaine)	GMP Cell Production	25 × 10^6^ MSC in 2 mL of saline 1 inj.	Intradiscal injection	MSCs group—VAS Baseline: 67 ± 26 3 mos: 43 ± 30 6 mos: 40 ± 29 12 mos: 47 ± 36 Placebo group—VAS Baseline: 62 ± 23 3 mos: 46 ± 27 6 mos: 51 ± 29 12 mos: 47 ± 28	-	MSCs group Baseline: 34 ± 23 3 mos: 16 ± 20 6 mos: 20 ± 24 12 mos: 22 ± 24 Placebo group Baseline: 24 ± 14 3 mos: 25 ± 15 6 mos: 30 ± 20 12 mos: 34 ± 25	Temporary pain
[46]	Allogeneic MSCs Placebo	GMP Cell Production	2 mL 1 inj.	Intradiscal injection	VAS Baseline 59.2 ± 16.75 1 mos: MSCs: 49.24 ± 24.33 Placebo: 49.80 ± 22.65 3 mos: MSCs: 45.33 ± 25.98 Placebo: 47.29 ± 23.55 6 mos: MSCs: 39.72 ± 25.87 Placebo: 41.98 ± 24.29 12 mos: MSCs: 33.68 ± 27.20 Placebo: 43.06 ± 25.12 24 mos: MSCs: 31.96 ± 25.02 Placebo: 34.41 ± 23.67	-	Baseline 29.9 ± 12.9 1 mos MSCs: 25.08 ± 16.67 Placebo: 27.81 ± 14.95 3 mos MSCs: 21.45 ± 16.07 Placebo: 23.92 ± 15.68 6 mos MSCs: 18.70 ± 13.42 Placebo: 22.59 ± 15.56 12 mos MSCs: 16.76 ± 14.50 Placebo: 21.08 ± 15.63 24 mos MSCs: 16.23 ± 16.07 Placebo: 19.41 ± 15.43	LBP worsening (n = 3 MSCs and n = 5 Placebo)
[38]	Autologous BMAC	Bone marrow concentration system	2–3 mL 1 inj.	Intradiscal injection	VAS (mean ± SE): Baseline: 82.1 ± 2.6 3 mos: 27 ± 5 6 mos: 18.7 ± 5 12 mos: 28.1 ± 7 24 mos: 22.9 ± 6	-	(mean ± SE) Baseline: 56.7 ± 3.6 3 mos: 19.9 ± 5 6 mos: 19 ± 4 12 mos: 22.3 ± 7 24 mos: 18.3 ± 6	n = 5 pts LBP worsening
[39]	Autologous BMAC	Bone marrow concentration system	2–3 mL 1 inj.	Intradiscal injection	VAS (mean ± SE): Baseline: 82.1 ± 2.6 3 mos: 27 ± 5 6 mos: 18.7 ± 5 12 mos: 28.1 ± 7 24 mos: 22.9 ± 6 36 mos: 21.9 ± 4.4	-	(mean ± SE) Baseline: 56.7 ± 3.6 3 mos: 19.9 ± 5 6 mos: 19 ± 4 12 mos: 22.3 ± 7 24 mos: 18.3 ± 6 36 mos: 17.5 ± 3.2	n = 6 pts LBP worsening
[47]	Autologous PRP Steroid	PRP obtained by two-spin technique	3–5 mL 1 inj.	Transforaminal injection	VAS: Baseline 6.7 ± 1.2 1 mos: 4.7 ± 1.5 3 mos: 3.9 ± 1 6 mos: 3.5 ± 1.4	-	Modified-ODI Baseline: 57.3 ± 9.7 1 mos: 42.5 ± 14.8 3 mos: 36.2 ± 9.7 6 mos: 33.3 ± 10.3	None
[48]	Autologous PRP Saline	Smart PReP 2 procedure	1 mL 1 inj.	Intradiscal injection	NRS: For average pain, 48% (95% CI: 34–62) in the PRP group achieved ≥2-point improvement vs. 35% (95% CI: 23–50) in placebo. For worst pain, 36% (95% CI: 23–51) in PRP vs. 40% (95% CI: 27–55) in placebo	Score Change (0–12 mo): PRP group: 12.7 → 9.6 (∆ = −3.1); Placebo group: 13.4 → 10.1 (∆ = −3.3)	-	Spondylodiscitis after PRP treatment
[49]	Autologous PRP Contrast agent	Standardized preparation	1–2 mL 1 inj.	Intradiscal injection	NRS Current Pain Baseline Placebo: 4.61 ± 2.21 Baseline PRP: 4.74 ± 2.21. 1 wk Placebo: 4.78 ± 1.99 1 wk PRP: 4.21 ± 1.99 1 mos Placebo: 4.61 ± 2.21 1 mos PRP 4.00 ± 2.21 2 mos Placebo: 4.39 ± 2.59 2 mos PRP: 3.09 ± 2.59 6 mos PRP: 3.60 ± 2.49 12 mos PRP: 3.15 ± 2.38 Best Pain Baseline Placebo: 2.08 ± 1.74 Baseline PRP: 2.81 ± 1.78 1 wk Placebo: 2.44 ± 1.82 1 wk PRP: 2.88 ± 1.83 1 mos Placebo: 2.28 ± 1.82 1 mos PRP: 2.53 ± 1.83 2 mos Placebo: 2.72 ± 2.12 2 mos PRP: 2.00 ± 2.06 6 mos PRP: 2.00 ± 2.33 12 mos PRP: 2.10 ± 2.20 Worst Pain Baseline Placebo: 7.72 ± 1.53 Baseline PRP: 7.98 ± 1.56 1 wk Placebo: 7.39 ± 1.95 1 wk PRP: 6.86 ± 1.94 1 mos Placebo: 7.11 ± 1.91 1 mos PRP: 6.41 ± 1.88 2 mos Placebo: 6.83 ± 2.33 2 mos PRP: 5.82 ± 2.33 6 mos PRP: 6.32 ± 2.12 12 mos PRP: 5.86 ± 2.20	-	-	None
[40]	Autologous PRP	Standardized preparation	2 mL 1 inj.	Intradiscal injection	NRS Baseline: Current pain: 31 ± 5.6 Best pain: 31 ± 4.1 Worst pain: 31 ± 6.9 12 mos: Current pain: 3.4 ± 1.4 Best pain: 3.0 ± 1.9 Worst pain: 4.6 ± 1.8	-	-	n = 1
[30]	Autologous PRP	Double spin technique	2 mL 1 inj.	Intradiscal injection	NPRS Clinically meaningful improvement based on NPRS alone: −38% of patients in the control group −22% of patients in the PRP group	-	Clinically meaningful improvement based on ODI alone: −38% of patients in the PRP group −39% of patients in the control (saline) group	NR

Abbreviations: VAS: Visual Analog Scale; NRS: Numeric Rating Scale; NPS: Numeric Pain Scale; RDQ: Roland Disability Questionnaire; RMDQ: Roland–Morris Disability Questionnaire; ODI: Oswestry Disability Index; FU: follow-up; CS: corticosteroid; PRGF: plasma rich in growth factors; ADSCs: adipose-derived mesenchymal stem cells; TEAEs: treatment-emergent adverse events; GMP: good manufacturing practice; MPCs: allogenic mesenchymal precursor cells; HA: hydroxyapatite; BMSCs: bone marrow mesenchymal stem cells; BMAC: bone marrow aspirate concentrate; LBP: low back pain; pts: patients.

**Table 5:** Imaging findings, quality of life measures, study design features, and declarations.

Ref.	MRI (Pre-op Pfirrmann Grade)	Radiological Findings (Pre-op Modic/Disc Height)	QoL (SF-36/EQ-5D, SF-12, NASS)	Subsequent Surgery (%)	Randomization	Blinding	Conflict of Interest	Funding Declared
[41]	Grade 3: 12 pts Grade 4: 2 pts	T2 value of the NP: Baseline: 0.56 ± 0.1 3–4 months: 0.53 ± 0.06 12 months: 0.56 ± 0.10 T2 value of the AF: Baseline: 1.01 ± 0.06 3–4 months: 1.01 ± 0.15 12 months: 0.94 ± 0.09	-	NR	None	NR	None	NR
[50]	PRP: Grade 4: 8 discs Grade 5: 0 discs Grade 6: 3 discs CS: Grade 4: 5 discs Grade 5: 3 discs Grade 6: 2 discs	-	-	NR	1:1	NR	None	Yes
[31]	Grade 3: 40.7% Grade 4: 20.3%	NR	-	NR	None	NR	Yes	None
[32]	-	Modic type I or II changes at the same level	EQ-5D-3L Baseline: 100% reported some difficulties (e.g., work, study, housework, family, leisure) 6 months: 44% (n = 4) no difficulties 12 months: 66% (n = 6) no difficulties	NR	None	NR	Yes	Yes
[42]	Grade 2: 1 pts Grade 3: 66 pts Grade 4: 219 pts Grade 5: 63 pts Grade 6: 54 pts Grade 7: 1 pts	-	EQ-5D-5L Baseline: MPCs: 0.66 ± 0.13 MPCs + HA: 0.66 ± 0.12 Control group: 0.67 ± 0.12 6 mos: MPCs: 0.75 ± 0.89 MPCs + HA: 0.74 ± 0.77 Control group: 0.73 ± 0.72 12 mos: MPCs: 0.74 ± 0.83 MPCs + HA: 0.76 ± 0.91 Control group: 0.72 ± 0.57 24 mos: MPCs: 0.74 ± 0.77 MPCs + HA: 0.73 ± 0.65 Control group: 0.72 ± 0.55 36 mos: MPCs: 0.75 ± 0.76 MPCs + HA: 0.75 ± 0.79 Control group: 0.71 ± 0.41	NR	1:1:1	Double-blind	Yes	Yes
[33]	-	-	-	n = 2	None	None	Yes	Yes
[34]	-	-	SF-12 Baseline: 56.15 ± 24.03	-	None	None	None	Yes
[43]	-	-	SF-12 PRP Baseline: 35 1 week: 45 3 wks: 55 6 wks: 65 SF-12 Steroid Baseline: 35 1 week: 50 3 wks: 55 6 wks: 52	NR	1:1	Double-blind	None	NR
[35]	Grades 1–2: 2 pts Grades 3–5: 30 pts	No Modic changes: 12 Modic 1–2: 20	EQ-5D-5L Baseline: 0.7 ± 0.1 12 mos: 0.76	n = 3	None	None	Yes	NR
[36]	-	-	-	NR	None	None	NR	NR
[37]	-	Type 1 or type 2 Modic changes	-	NR	None	NR	NR	NR
[44]	-	-	NASS Placebo: Baseline: 3.1 ± 0.5 1 mos: 2.8 ± 0.5 6 mos: 2.6 ± 0.5 12 mos: 2.5 ± 0.5 PRP: Baseline: 3.1 ± 0.5 1 mos: 2.4 ± 0.4 6 mos: 2.1 ± 0.4 12 mos: 1.8 ± 0.4 BMC Baseline: 3.2 ± 0.5 1 mos: 2.1 ± 0.4 6 mos: 1.8 ± 0.4 12 mos: 1.7 ± 0.4	None	Yes	NR	NR	NR
[45]	Grade 2, 3, 4	-	SF-12—MCS MSCs: Baseline: 46 ± 3 3 mos: 50 ± 2 6 mos: 52 ± 2 12 mos: 48 ± 3 Placebo: Baseline: 52 ± 3 3 mos: 46 ± 3 6 mos: 48 ± 3 12 mos: 50 ± 3 SF-12—PCS MSCs: Baseline: 39 ± 2 3 mos: 47 ± 3 6 mos: 46 ± 3 12 mos: 45 ± 3 Placebo: Baseline: 40 ± 3 3 mos: 43 ± 3 6 mos: 39 ± 3 12 mos: 42 ± 3	NR	Yes	Yes	No	Yes
[46]	Grade: 4–7	-	SF-36 Baseline: PCS: 37.2 (32.2; 42.0) MCS: 38.2 (33.9; 47.7)	None	1:1	Yes	None	Yes
[38]	Grade 6–7 at one or two levels	-	-	n = 5	No	Yes	NR	NR
[39]	Grade 4–7 at one or two levels	-	-	n = 6	No	Yes	NR	No
[47]	Grade: 2–3	-	-	None	Yes	Double-blind	No	No
[48]	-	No Modic changes	SF-12—PCS Baseline vs. 1 year: −1.19; 95% CI −5.39 to 2.99 SF-12—MCS Baseline vs 1 year: −0.34; 95% CI −3.99 to 3.29	n = 2	1:1	Single-blinded	No	NR
[49]	-	-	SF-36 Pain Baseline Placebo: 47.92 ± 21.13 Baseline PRP: 43.28 ± 21.11 1 wk Placebo: 47.22 ± 21.76 1 wk PRP: 40.52 ± 21.76 1 mos Placebo: 47.22 ± 19.98 1 mos PRP: 55.17 ± 19.98 2 mos Placebo: 52.78 ± 22.19 2 mos PRP: 61.29 ± 22.19 6 mos PRP: 57.95 ± 25.45 12 mos PRP: 67.79 ± 23.51 SF-36 Physical Function Baseline Placebo: 56.11 ± 18.54 Baseline PRP: 56.40 ± 18.52 1 wk Placebo: 51.28 ± 20.04 1 wk PRP: 51.63 ± 20.46 1 mos Placebo: 60.97 ± 21.43 1 mos PRP: 58.43 ± 21.17 2 mos Placebo: 57.08 ± 22.91 2 mos PRP: 61.70 ± 22.89 6 mos PRP: 67.14 ± 24.18 12 mos PRP: 73.20 ± 19.38	None	2:1	Double-blind	Yes	Yes
[40]	Grade 3: 21 pts Grade 4: 10 pts	-	SF-36 Baseline: Pain score: 45.0 ± 13.1 PCS: 51.8 ± 13.1 12 mos: Pain score: 66.8 ± 18.1 PCS: 67.7 ± 14.4	n = 1	No	No	No	Yes
[30]	Grade ≤ 4	-	-	NR	2:1	Double-blind	No	No

Abbreviations: QoL: quality of life; NP: nucleus pulposus; AF: annulus fibrosus; MCS: mental component summary; PCS: physical component summary; NR: not reported.

## Data Availability

The data that support the findings of this study are available from the corresponding author upon reasonable request.

## Abbreviations

The following abbreviations are used in this manuscript:

PRP	Platelet-rich plasma
MSC	Mesenchymal stem cells
DDD	Degenerative disc disease
RCT	Randomized controlled trials
MD	Mean difference
ODI	Oswestry Disability Index
LBP	Low back pain
MRI	Magnetic resonance imaging
CT	Computed tomography
NSAIDs	Non-steroidal anti-inflammatory drugs
PDGF	Platelet-derived growth factor
TGF-β	Transforming growth factor-beta
VEGF	Vascular endothelial growth factor
BMSCs	Bone marrow mesenchymal stem cells
ADSCs	Adipose-derived mesenchymal stem cells
IGF-1	Insulin-like growth factor 1
NPC	Nucleus pulposus cell
ECM	Extracellular matrix
PRGF	Plasma rich in growth factors
MPCs	Mesenchymal precursor cells
BMAC	Bone marrow aspirate concentrate
VAS	Visual Analog Scale
NRS	Numeric Rating Scale
RMDQ	Roland–Morris Disability Questionnaire
SF-12	Short form-12
SF-36	Short form-36
EQ-5D	EuroQol-5D
IVD	Intervertebral disc
NPS	Numeric Pain Scale
FU	Follow-up
CS	Corticosteroid
TEAEs	Treatment-emergent adverse events
GMP	Good manufacturing practice
HA	Hydroxyapatite
pts	Patients
Qol	Quality of life
NP	Nucleus pulposus
AF	Annulus fibrosus
MCS	Mental component summary
PCS	Physical component summary
NR	Not reported
SD	Standard deviation
CIs	Confidence intervals
SMD	Standardized Mean Difference
